# Pathophysiology of ageing, longevity and age related diseases

**DOI:** 10.1186/1742-4933-4-4

**Published:** 2007-08-02

**Authors:** Alexander Bürkle, Graziella Caselli, Claudio Franceschi, Erminia Mariani, Paolo Sansoni, Angela Santoni, Giancarlo Vecchio, Jacek M Witkowski, Calogero Caruso

**Affiliations:** 1 Department of Biology, Box X911, University of Konstanz, Konstanz, Germany; 2 Department of Demographic Sciences, University "La Sapienza" of Rome, Rome, Italy; 3 Department of Experimental Pathology and CIG – Interdepartmental Center "L. Galvani", University of Bologna, Italy; 4 Immunology and Genetics Laboratory, Institute Codivilla-Putti, Bologna, Italy; 5 Department of Internal Medicine and Gastroenterology of University of Bologna, Bologna, Italy; 6 Department of Internal Medicine and Biomedical Sciences, University of Parma, Parma, Italy; 7 Department of Experimental Medicine, University "La Sapienza" of Rome, Rome, Italy; 8 Department of Cellular and Molecular Biology and Pathology, University of Naples Federico II, Naples, Italy; 9 Department of Pathophysiology, Medical University of Gdansk, Gdansk, Poland; 10 Immunosenescence Unit, Department of Pathobiology and Biomedical Methodologies, University of Palermo, Palermo, Italy

## Abstract

On April 18, 2007 an international meeting on Pathophysiology of Ageing, Longevity and Age-Related Diseases was held in Palermo, Italy. Several interesting topics on Cancer, Immunosenescence, Age-related inflammatory diseases and longevity were discussed. In this report we summarize the most important issues. However, ageing must be considered an unavoidable end point of the life history of each individual, nevertheless the increasing knowledge on ageing mechanisms, allows envisaging many different strategies to cope with, and delay it. So, a better understanding of pathophysiology of ageing and age-related disease is essential for giving everybody a reasonable chance for living a long and enjoyable final part of the life.

## Background

On April 18, 2007 an international meeting on Pathophysiology of Ageing, Longevity and Age-Related Diseases was held in Palermo, Italy. Several interesting topics were discussed. Here we summarize the most important issues.

### Cancer

The majority of cases of cancer occur in patients over the age of 65. Cancer rates increase sharply with age in both sexes: the incidence of cancer is 12–36 times higher in individuals aged 65 years or older than in individuals aged 25–44 years, and 2–3 times more common than in persons aged 45–64 years. It is worth noting that 70% of deaths attributable to all forms of cancer occur in men and in women aged 65 years or older, whereas 35% cancer deaths in men and 46% of cancer deaths in women occurred in those aged 75 years or older. The relationship between ageing and cancer is similar for most forms of cancer, and it is well described by the multistage model of carcinogenesis. Therefore ageing might be considered not as a determinant of cancer *per se*, but as a surrogate marker of the duration of exposure to relevant carcinogenic factors [1]. On the other hand the relationship between cancer and inflammation have recently been revised and it has been suggested that the inflammatory cells and cytokines found in tumours are more likely to contribute to tumour growth and progression [2]. Moreover cancer susceptibility and severity may be associated with functional polymorphisms of inflammatory cytokine genes [1]. If genetic damage is the "match that lights the fire" of cancer, some types of inflammation may provide the "fuel that feeds the flames" [2]. Thus, the increased frequencies of cancer in ageing might be due to well-known pro-inflammatory status of ageing [3].

In the Symposium, the role of oncogenes in human cancer was highlighted by analysing human epithelial thyroid tumours deriving either from follicular or from parafollicular (C) cells. Follicular-cell derived tumours represent a wide spectrum of lesions, ranging from benign adenomas through differentiated, papillary and follicular carcinomas, and undifferentiated, anaplastic, carcinomas, thus providing a good model for finding a correlation between specific genetic lesions and histologic phenotype. Follicular adenomas frequently show the presence of mutations in one of the three ras genes, *HRAS*, *KRAS*, and *NRAS*. G stimulatory protein (gsp) and thyrotropin receptor (TSH-R) mutations are at the origin of hyperfunctioning benign tumours (toxic nodules and adenomas). The two different types of differentiated thyroid carcinomas exhibit not only different morphology, but also different behaviour and are associated with mutations in different oncogenes: papillary carcinoma with rearrangements of either the *RET *or *TRK *genes and follicular carcinomas with mutations of one of the three ras oncogenes. The p53 tumour suppressor gene is frequently associated with anaplastic thyroid carcinomas. *RET *is a paradigmatic example of how different mutations of a single gene can lead to different neoplastic phenotypes. Somatic rearrangements, often caused by chromosomal inversions, activate the oncogenic potential of *RET *in human thyroid papillary carcinomas. These lesions occur in almost 50% of papillary cancers and consist in the juxtaposition of the 3' tyrosine kinase domain of the *RET *gene which codes for a receptor protein not normally expressed in follicular cells with the 5' domain of ubiquitously expressed genes, giving rise to several types of *RET/PTC*, papillary thyroid carcinomas, chimeric genes. Such ubiquitously expressed genes provide the promoter and dimerization functions, necessary for the constitutive activation of *RET/PTC *proteins. *RET *germline point mutations are responsible of familial multiple endocrine neoplasia type 2 syndromes (MEN 2) represented by (a) Familial Medullary Thyroid Carcinoma (FMTC), (b) MEN2A and (c) MEN2B, a common feature of which is the medullary thyroid carcinoma, a malignant tumour derived from parafollicular C-cells. *RET *point mutations can also occur as a somatic event in sporadic medullary thyroid carcinomas and pheochromocytomas. The detailed knowledge of the specific *RET *mutations responsible for human thyroid tumours provides relevant tools for the clinical management of these diseases [4,5].

### Immunosenescence

In the elderly, many alterations in innate and acquired immunity have been described and viewed as deleterious, hence the term immunosenescence. On the other hand, immunosenescence is a complex process involving multiple re-organisational and developmentally regulated changes, rather than simple unidirectional decline of the whole function. However, some immunological parameters are often reduced significantly in the elderly and, *vice versa *good function is tightly correlated to health status. Recent observations indicate that immunosenescence is not accompanied by an unavoidable and progressive deterioration of the immune function, but is rather the result of a remodelling where some functions are reduced while others remain unchanged or are even increased. Importantly, age-related changes of the immune system are directly or indirectly involved in the well-known susceptibility of the elderly to infectious diseases, autoimmunity, and cancer and in the decreased responsiveness to vaccination. The same is true for the pathogenesis of the more relevant age-related diseases, such as cardiovascular and neurodegenerative diseases, diabetes, and osteoporosis. In fact, all these diseases share an important immune component implicated in their pathogenesis. In addition it appears that the innate compartment of the immune system is relatively well preserved during ageing in comparison to the more recent and sophisticated clonotypic compartment that exhibits more profound modifications [3,6-8].

Senescence of clonotypic immunity is mostly the result of alterations to T cells. Lifelong chronic antigen load seems to be the major driving force of immunosenescence, which impacts on human lifespan by reducing the number of virgin, *i.e*. antigen-non experienced, T cells, and simultaneously fills the immunological space with expanded clones of memory and effector, *i.e*. antigen-experienced, T cells [9]. Such lifelong and chronic antigen load is responsible for the chronic inflammatory status that characterises ageing [3]. The progressive reduction of naïve T cells involving both CD4+ and CD8+ T lymphocytes is paralleled by a concomitant increase of memory CD28- T cells expressing a senescent phenotype, *i.e*. progressive shortening of telomeres and reduced replicative capacity [10]. A second fundamental aspect of immunosenescence is the progressive age-related increase of a proinflammatory status represented by an increase of inflammatory cytokines and of inflammatory markers predictive of mobility and mortality [11,12]. This pro-inflammatory condition is referred to chronic antigenic load (bacteria, virus, fungi, toxins, mutated cells) that continuously stimulate innate immunity and seems to favour the onset of typical age-related diseases (atherosclerosis, dementia, osteoporosis, neoplasia) where immune and autoimmune factors play an important role [3].

It has been suggested that chronic viral antigenic stimulation could be responsible of age-related modifications of lymphocyte subsets including clonal expansion of viral antigen-specific CD8+ T cells expressing a memory phenotype and that can represent up to a quarter of the whole CD8+ T cell population [13]. In a recent study [14], frequencies and phenotype of CD8+ T cell have been evaluated in nonagenarians and centenarians by HLA-A2 and B7 tetramers that incorporated epitopes specific for Epstein-Barr Virus (EBV) and Cytomegalovirus (CMV). The data demonstrate that EBV and CMV induce quantitatively and qualitatively different CD8+ T cell responses in advanced age. The frequency and absolute number of CD8+ T cells specific for three EBV-epitopes were relatively low and mostly included within CD8+CD28+ cells. By contrast, CMV infection was characterised by variable numbers of CD8+ T cells specific for two CMV epitopes that, in some subjects, were strikingly expanded and did not express the CD28 molecule. In order to further clarify the roles of CMV infection and the host, 121 subjects aged 25–100 were recently studied: 18 subjects were seronegative and 103 seropositive for CMV infection. It was observed that the age-dependent reduction of naïve CD8+ T cells was accelerated in CMV+ subjects while the decline of naïve CD4+ T cells was not modified by the CMV infection. The reduction of naïve CD8+ cells was accompanied by a progressive increase of effector CD8+CD28- cells particularly in CMV+ subjects. An age-dependent accumulation of CD4+CD28- cells was only observed in CMV+ subjects while these cells were virtually absent within CMV- subjects. Samples of peripheral blood mononuclear cells were stimulated with mixtures of peptides spanning the entire pp65 and IE-1 CMV protein and thereafter the responding cells, Interferon(IFN)-γ+, were recorded both among CD8+ and CD4+ T cells. At the functional level, an age-dependent accumulation of CMV-specific (IFN-γ+) CD8+ cells was observed while an increase of pp65-specific CD4+ cells was only observed within subjects over 85 years of age. The majority of CMV-specific CD8+ (IFN-γ+) and 25% of CD4+ (IFN-γ+) expressed the cytotoxic degranulation marker CD107a (Sansoni et al., paper submitted). These data support the view that chronic CMV infection is responsible for profound modifications of the lymphocyte subsets not only involving CD8+ but also CD4+ T cells and probably concurring to the age-dependent proinflammatory status that accompanies the main of age-associated diseases.

The study of the human immune response in healthy elderly donors has shown that immunosenescence not only affects the T cell response, but also various aspects of innate immunity, and probably the most extensive studies on age associated alterations in the innate immune system have been directed toward Natural Killer (NK) cells. NK cells, together with polymorphonuclear cells and macrophages, are components of the natural immune system. They represent a primary host defence system because they are responsible for spontaneous killing of certain tumour cells and of virally infected cells. NK cells lack T cell receptors and are characterised by the membrane expression of CD56 and CD16. In addition, they display two alternative mechanisms of cytolysis: a direct spontaneous cytotoxicity against a variety of tumour cells and indirect Fc receptor-mediated cytotoxicity against antibody-coated targets (Antibody-Dependent Cell-mediated Cytotoxicity; ADCC). [15]. A balance of signals received from multiple activating and inhibitory receptors regulates their effector functions. These receptors allow NK cells to rapidly survey their environment for potentially dangerous cells. When an imbalance in signalling favours activation, secretion of cytokines and/or release of cytotoxic granules occurs. In humans, NKG2D is one of the activating receptors that is expressed on NK cells, Tγδ cells, and CD8αβ T cells. NKG2D recognises as ligands UL16-binding protein 1 (ULBP1), ULBP2, ULBP3, ULBP4, and the MHC class I chain-related molecules, MICA and MICB. These NKG2D ligands are generally absent or expressed at low levels on most healthy cells, but can be induced by viral and bacterial infections. Several studies have focused on the ability of NK cells to regulate acquired immune responses through the production of Th1-type cytokines early during infection or through the activation of dendritic cells. In addition, by establishing co-cultures of NK- and antigen-activated T cells, it has been shown that human NK cells can be induced to secrete IFN-γ in response to Interleukin(IL)-2 produced by activated T cells. In contrast, much less has been reported about the physical interactions that may take place between NK cells and immune cells of acquired branch of the response, in particular CD4^+ ^T cells. NK cells promote acquired immune responses through their production of type 1 and type 2 cytokines or chemokines. Secretion of these factors by activated NK cells influences the differentiation of B and T lymphocytes. Increasing evidence indicates that NK cells are also directly involved in dendritic cell maturation. By contrast, a potential role for direct cell-cell interactions between NK and T lymphocytes, in particular CD4^+ ^T cells, has not been explored. It has provided evidence that activated human NK cells are able of promoting TCR-dependent proliferation of resting autologous peripheral blood CD4^+ ^T cells by a process that involves co-stimulatory molecules of the immunoglobulin and tumour necrosis factor (TNF) superfamilies. These findings suggest a novel link between natural and acquired immune responses [16,17].

In 1987 a quantitative analysis of cells expressing the NK phenotype demonstrated that circulating NK cells increase in the peripheral blood of healthy individuals over 70 years old, compared to young or middle-aged people [18]. The increase in NK cell numbers in the peripheral blood from elderly people is directly correlated with age and with a decreased number of T cells supporting the idea of a compensative increase in NK cells for a decreased cytolytic activity. The cytolytic activity displayed by peripheral blood lymphocytes must be roughly dependent on the relative proportion of the NK cells present in the sample. By contrast the cytolytic activity of NK cells following incubation with K562 cells was found to be similar between the elderly, extremely healthy subjects selected according to the SENIEUR protocol and young subjects, despite the twofold increased number of effector cells [18]. In any case, purified or cloned populations of NK cells showed a decreased cytolytic activity in the aged on a per cell basis. This was consistent with the finding that, following target binding, each CD16-positive cell from elderly donors exerts only about half of the cytolytic activity displayed by NK cells from young donors [19]. However, neither the binding of effector to target cells nor the effector cells content, distribution or utilisation of perforin were significantly different between young and old groups. Therefore some other factors must account for the decreased lytic activity found in NK cells from aged people. In fact, the ability of NK cells to transform a receptor-mediated signal into an effector response, which is linked to the ability to generate second messengers after K562 stimulation, does show a pronounced age-related decrease. The major biochemical defect appears to be an age-related delay in PIP_2 _hydrolysis and decrease in the levels of IP_3 _formation in old NK cells after K562 stimulation [20]. Since an essentially preserved density of surface receptors involved in recognition and adhesion on NK cells was observed during ageing and also a preserved ability of NK cells to form conjugates with target cells, signal transduction may be impaired at a step distal to the binding of receptors.

Increasing evidence has demonstrated that the immune, endocrine and nervous systems are closely integrated and communicate through circulating cytokines, hormones and neurotransmitters. Many hormones and micronutrients have an important influence on the homeostasis of the immune system and in maintaining the integrity of body composition. The age-related decrease in adipose tissue, muscle and bone mass together with an increased risk of malnutrition, vitamin and trace element deficiency are among the major factors implicated in the development of frailty syndromes and impaired resistance to infections in the elderly. NK cell number and cytolytic activity were significantly associated with serum concentrations of vitamin D, and this association was consistent with the evidence that vitamin D supplementation in elderly subjects *in vivo *significantly modulated NK activity, increasing circulating levels of IFN-α. Anthropometric parameters indicating fat reserves and muscle reserves were also directly correlated with NK cell number and activity, and fat indicators were linked to vitamin D [21]. A further remarkable finding is the strong correlation between the number of NK cells and serum concentrations of zinc, a key element in many homeostatic responses of the body, including oxidative stress and in many biological functions, including immune efficiency [22]. In addition, zinc-aspartate supplementation in subjects with low or borderline normal circulating zinc levels increased concentration of this ion and was able to up-modulate NK cytolytic activity (Mariani, unpublished observations), suggesting a conversion of the pro-inflammatory status (characterised by high levels pro-inflammatory cytokines and possibly chemokines) [23] into more balanced Th1/Th2 responses. Because of the close correlation between the degree of malnutrition and immunodeficiency in the elderly (increasing the risk of infection, as demonstrated by the high number of non-responders among undernourished aged subjects who received influenza vaccination), these results stress the paramount importance of nutritional evaluation in the clinical assessment of elderly people ([24], for an extensive review).

### Age-related inflammatory disease

The individual rates of ageing for the whole organism or for any organ system may vary, depending on genetics, disease history, chance, etc. This is especially true for the immune system. Impaired homeostasis and function of the immune system (especially of the CD4^+ ^lymphocytes as the hub immune cells) underlies or at least participates in the pathology of Alzheimer's disease (AD) and of rheumatoid arthritis (RA). Both diseases belong to those that accelerate ageing (shorten the lifespan) of the sufferers. The question arising is therefore if the CD4^+ ^cells of RA and/or AD patients undergo an accelerated ageing themselves. The major functionalities of the CD4^+ ^lymphocytes are manufacturing of multitude of cytokines and periodical proliferation to make effector and memory clones. The CD4^+ ^lymphocytes of RA patients are already known to exhibit certain features similar to those observed for these cells in the healthy elderly, including relatively short telomeres, decreased amount of surface CD28, decreased overall proliferation etc. To investigate the possibility of accelerated aging of CD4^+ ^lymphocytes of RA and AD patients, a new flow-cytometric technique of studying lymphocyte proliferation has been utilised. This technique is, based on staining with Carboxyfluorescein Succinimidyl ester and extensive mathematical analysis of acquired data, that allows for better quantification of proliferating lymphocytes, as well as calculation of dynamic parameters of proliferation including the timing of the cycle itself and of the G0→ G1 transition time. It allowed to show that CD4^+ ^cells of RA patients (especially younger ones) do not differ in these respects form those of healthy elderly, corroborating with the idea of their accelerated ageing. At least one of these parameters (the length of the G0→ G1 transition phase) seems to be correlated with the level of CD28 expression of the lymphocytes' surface which, in turn, depends on the regulatory activity of the proinflammatory cytokine TNF. Also another gene, Klotho) (sometimes dubbed the 'ageing hormone') contains a putative TNF-responsive regulatory sequence. Following this trace it has been demonstrated that both the transcriptional activity of the gene and the cellular contents of Klotho protein is greatly reduced in the CD4^+ ^cells of RA patients regardless from their chronological age, and similar to that seen in the cells of healthy elderly. Expectedly, an enzymatic β-glucuronidase activity ascribed to Klotho protein (supposedly involved in the hydrolysis of steroid glucuronides) is reduced in both the RA and healthy elderly CD4^+ ^lymphocytes, which may participate in the pro-inflammatory status of both elderly and the RA patients [25,26]. Using the same methodology for the CD4^+ ^cells of AD patients the quasi-opposite change was found. Namely, these lymphocytes show the values of the dynamic parameters of proliferation *in vitro*, including the cell cycle and the G0∋ G1 time resembling these of the cells from healthy young individuals, despite being drawn from typical, elderly AD sufferers. Apparently, the exposure to β-amyloid peptides seems to play the role in these different results obtained for AD cells, especially the numbers of divisions, proliferation index and the G0→ G1 time; interestingly, the CD4^+ ^cells of AD patients seem to react to the peptides more than these of healthy ones, suggesting possible role of different genetic background (the HLA?) as one of the reasons for difference. In summary, these data show that RA may be considered a case for accelerated aging of the CD4^+ ^lymphocytes, while AD is not, although AD lymphocytes do display a deviation from normal function (Witkowski, unpublished observations).

### DNA repair

Half a century ago, when the free radical theory of aging was first proposed, the damaging effects of reactive oxygen species (ROS) were in the focus of attention and considered the single most important determinant of aging. Two decades later, however, the disposable soma theory of ageing redirected the attention to the potential impact of cellular maintenance and repair pathways that are both genetically and environmentally determined and are counteracting the damaging effects of ROS. In this context poly(ADP-ribosyl)ation, a DNA-damage driven posttranslational modification of proteins, is of particular interest. Poly(ADP-ribosyl)ation is catalysed by poly(ADP-ribose) polymerase-1 (PARP-1), with NAD^+ ^serving as substrate [27]. PARP-1 activation is triggered by DNA strand breaks, is functionally associated with DNA repair pathways and is a survival factor for cells under low to moderate levels of genotoxic stress. Over a decade ago, a positive correlation between poly(ADP-ribosyl)ation capacity of mononuclear blood cells with longevity of mammalian species was described [28]. The subsequent comparison of purified recombinant human and rat PARP-1 revealed that this correlation might be explained in part by evolutionary sequence divergence [29]. This view fits perfectly with recent results from the literature on different knockout mice defective for nucleotide excision repair genes, revealing a crucial role of DNA repair as a longevity assurance pathway. In order better to understand the role of DNA repair and of poly(ADP-ribosyl)ation in ageing, the Bürkle group has recently developed an improved method to quantify DNA strand breaks and DNA crosslink formation in living cells by an automated fluorescence-detected alkaline DNA unwinding (FADU) assay. They have also set up a new assay to monitor poly(ADP-ribose) formation in living cells by flow cytometry, based on an assay previously developed for permeabilised cells [30].

### Longevity

The improvements of the social-environmental conditions, of medical care, and the quality of life caused a general improvement of the health status of the population and a consequent reduction of the overall morbidity and mortality, resulting in an overall increase of life expectancy. Around the 1970s, the progressive decline of mortality (1–2% per year) in individuals over 80 years old has increased in all industrialized countries, so that the number of centenarians has augmented about 20-fold. Centenarians represent a cohort of select survivors who benefit from a delay in the onset of diseases that often cause mortality in the general population at significantly younger ages [31]. A body data collected on the genetics of human longevity, mostly resulting from studies on centenarians, indicates that the following: Centenarians and long-living sibpairs are a good choice for the study of human longevity, because they represent an extreme phenotype, *i.e*. the survival tail of the population who escaped neonatal mortality, pre-antibiotic era illnesses, and fatal outcomes of age-related complex diseases. The model of centenarians is not simply an additional model with respect to well-studied organisms, and the study of humans has revealed characteristics of ageing and longevity (geographical and gender differences, role of antigenic load and inflammation, role of mtDNA variants) which did not emerge from studies in animal model systems. All the phenotypic characteristics of nonagenarians and centenarians fit the hypothesis that ageing is a remodelling process where the body of survivors progressively adapts to internal and external damaging agents they are exposed to during several decades, largely unpredicted by evolution. Such a remodelling process, which can be considered a Darwinian process occurring at the somatic level within the framework of the evolutionary constraints established by evolution for *H. sapiens *as a species may explain why the same gene polymorphism can have different (beneficial or detrimental) effects at different ages. Demographic evidence suggests that longevity can be achieved by different combinations of genes, environment, and chance, in a pattern that may be quantitatively and qualitatively different in different geographic areas, and that population-specific genetic factors play a role in the longevity phenotype. The concomitant and integrated use of new *in silico *and high-throughput strategies will greatly accelerate the identification of new longevity genes in humans [32-36,3].

**Figure 1:**
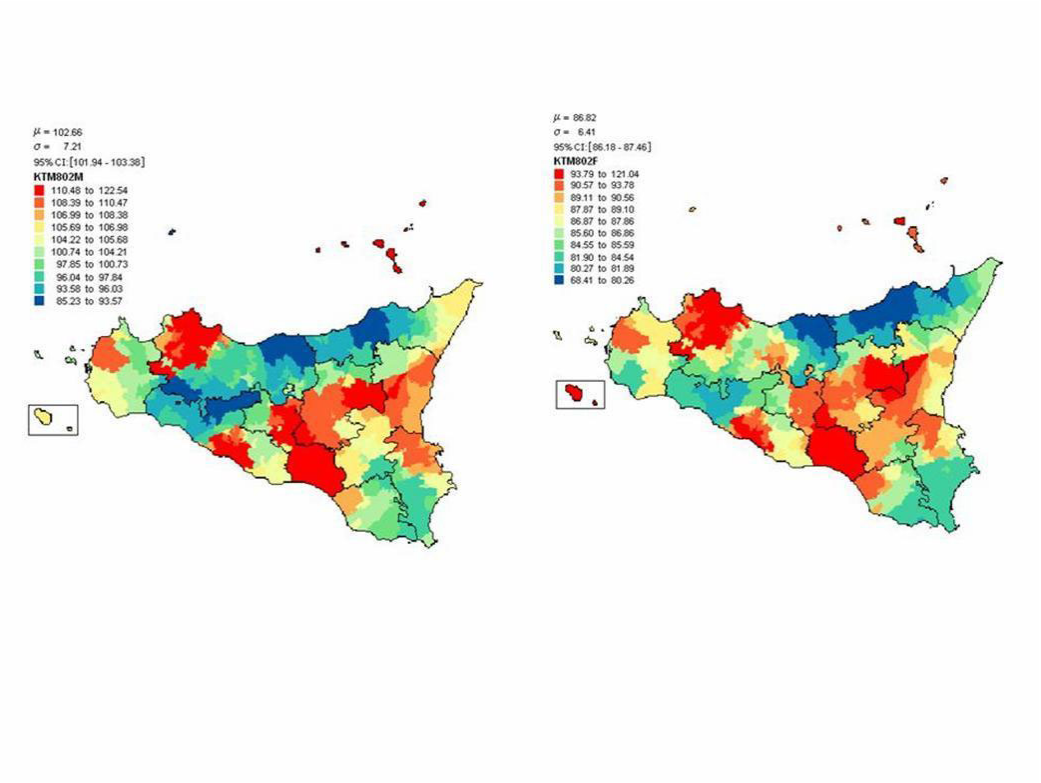
Mortality in Sicily after age of 80 years: Male (left) Female (right) period 1994–2001 (low mortality in blue, high mortality in red). For women in blue area the mortality is higher than in Italy. The municipalities concerned do not include polluted areas and are small, with the lowest number of inhabitants.

There is a widespread consensus that the existence of a greater or a lesser number of centenarians largely depends on mortality features between 80 and 100 years of age. In fact, lower mortality among this age group implies that more people should live until they are aged 100 or over. Hence, demographers apply an indicator of mortality between 80 and 100 years in order to define the longevity of a population and not, as is done elsewhere, the proportion of centenarians in the population. Sardinia, two second largest island of Italy, has drawn widespread interest because of the large number of centenarians and the existence of a geographical area where male mortality after 80 years is lower than anywhere else in the region and Italy [37,38]. This area covers various municipalities in the centre of the island, to the south of the province of Nuoro, where male mortality from cardiovascular diseases and cancers is especially low [39]. Focussing on genetically isolated populations, who are more homogeneous than the overall population with respect to cultural and historical background, origin and demographic parameters, is considered the most promising tool for the analysis and mapping of continuous multifactorial traits [40]. Once having seen the interesting outcome for Sardinia, it has turned focus to the largest island, Sicily, to see if something similar exists. The first aim was to identify geographic areas that are homogeneous with regard to low mortality of men and women after age 80 and to investigate the region-specific causes of death after this age. The second aim was to compare Sicily and Sardinia to see if there are some analogies and to find an explanation. Reference periods were 1981–1990 and 1991–2001. The 2001 census reports 390 municipalities in Sicily and 377 in Sardinia. The 386 and 363, respectively, selected here are geographically similar at the starting date of the municipal analysis (1981). Standardized mortality ratios (SMR) were calculated by municipalities (for all-cause and specific cause mortality) for ages 80 and over according to procedures applied in epidemiology. The Kernel density estimator is used to construct geographical maps. Kernel density estimators are the smoothed SMRs obtained as the spatial moving average for a number of municipalities adjacent to a given municipality. Results obtained highlight an area of longevity for men but not for women in Sicily with similar features to those found in Sardinia (Figure 1). In both cases the municipalities concerned do not include polluted areas and are small, with the lowest number of inhabitants. So, longevity concerns men living in a small town, without pollution, likely because of different working conditions, different life style *i.e*. reduced smoking and alcohol abuse and Mediterranean diet. Accordingly, these areas both in Sicily and in Sardinia also share low mortality from cancers and cardiovascular diseases. (Caselli & Lipsi, unpublished observations). Longevity is reduced for women likely because of different women condition and different educational level which produce a different access to prevention or to health facilities. The reason because longevity has been observed particulary in small municipalities is not surprising. It is well established, in fact, that individuals with greater access to social support and family network have better health and lower levels of mortality, particularly when adult daughters are present.

### Conclusive remarks

To conclude, ageing must be considered an unavoidable end point of the life history of each individual, nevertheless the increasing knowledge on ageing mechanisms, allows envisaging many different strategies to cope with, and delay it. So, a better understanding of pathophysiology of ageing and age-related disease is essential for giving everybody a reasonable chance for living a long and enjoyable final part of the life.

## Competing interests

The author(s) declare that they have no competing interests.

## Authors' contributions

AB carried out the molecular genetic studies on DNA damage, GC performed the demographic studies, CF and CC conceived the study on Ageing, longevity and inflammation, EM focused the attention on Cytokines and Chemokines, PS studied CD8+ and immunodeficiency in ageing, AS carried out assays on NK receptors and signalling, JMW on CD4+ and AD and RA and GV *RET *mutations in human thyroid tumours. All authors read and approved the final manuscript version.
